# An Excreta Cage

**Published:** 1895-05-25

**Authors:** 


### EDITOR'S LETTER-BOX.

[Our correspondents are reminded that proxility is a great bar to publication, and that brevity of style and conciseness of statement greatly facilitate early insertion.]

### AN EXCRETA CAGE.

Mr. Newton H. Nixon, Secretary of University College Hospital, writes: I have read your article in The Hospital of the 18th inst. upon the new Adelaide Hospital, and in which the following paragraph occurs: "The construction of a ventilated 'fæcal cupboard' is the result of a suggestion made by Professor Allen, of Melbourne University, and is, we may assume, a practical outcome of his recent visit to English hospitals." I would venture to point out that the first "fæcal cupboard" in use in England was erected in this hospital upon my suggestions and drawings, and a cupboard is now attached to each sanitary block. The invention was exhibited by Messrs. Mayer and Meltzer, of Great Portland Street, at the International Medical and Sanitary Congress in 1882, where it was awarded a Certificate of Merit, and also at the National Health Society's Exhibition, 1883, when a Silver Medal was awarded.

*** We agree with Mr. Nixon that most probably Professor Allen adopted his invention of a ventilated excreta cage, a most favourable notice of which appeared in our columns on its introduction.—Ed. *T. H.*

